# Dealing with spatial misalignment to model the relationship between deprivation and life expectancy: a model-based geostatistical approach

**DOI:** 10.1186/s12942-020-00200-w

**Published:** 2020-03-04

**Authors:** Olatunji Johnson, Peter Diggle, Emanuele Giorgi

**Affiliations:** CHICAS Research Group, Lancaster Medical School, Lancaster University, Bailrigg, Lancaster, UK

**Keywords:** Deprivation, Life expectancy, Likelihood-based inference, Model-based geostatistics, Spatial misalignment, Health inequality

## Abstract

**Background:**

Life expectancy at birth (LEB), one of the main indicators of human longevity, has often been used to characterise the health status of a population. Understanding its relationships with the deprivation is key to develop policies and evaluate interventions that are aimed at reducing health inequalities. However, methodological challenges in the analysis of LEB data arise from the fact that different Government agencies often provide spatially aggregated information on LEB and the index of multiple deprivation (IMD) at different spatial scales. Our objective is to develop a geostatistical framework that, unlike existing methods of inference, allows to carry out spatially continuous prediction while dealing with spatial misalignment of the areal-level data.

**Methods:**

We developed a model-based geostatistical approach for the joint analysis of LEB and IMD, when these are available over different partitions of the study region. We model the spatial correlation in LEB and IMD across the areal units using inter-point distances based on a regular grid covering the whole of the study area. The advantages and strengths of the new methodology are illustrated through an analysis of LEB and IMD data from the Liverpool district council.

**Results:**

We found that the effect of IMD on LEB is stronger in males than in females, explaining about 63.35% of the spatial variation in LEB in the former group and 38.92% in the latter. We also estimate that LEB is about 8.5 years lower between the most and least deprived area of Liverpool for men, and 7.1 years for women. Finally, we find that LEB, both in males and females, is at least 80% likely to be above the England wide average only in some areas falling in the electoral wards of Childwall, Woolton and Church.

**Conclusion:**

The novel model-based geostatistical framework provides a feasible solution to the spatial misalignment problem. More importantly, the proposed methodology has the following advantages over existing methods used model LEB: (1) it can deliver spatially continuous inferences using spatially aggregated data; (2) it can be applied to any form of misalignment with information provided at a range of spatial scales, from areal-level to pixel-level.

## Background

Over the last decades, access to better healthcare and education have led to a surge in human longevity, especially in high-income countries [1–3]. Life expectancy at birth (LEB), one of the main indicators of human longevity, has often been used to characterise the health status of a population [4]. Measuring deprivation is also important in order to describe health inequalities within a population and to better understand variation in health outcomes [5, 6]. Previous studies have shown that the LEB is strongly affected by deprivation [2, 7, 8] and that differences in LEB between most and least deprived individuals are larger among men than women [9, 10].

The main determinants of human longevity can be generally classified into social factors, genetic traits, life-style (e.g. consumption of tobacco, alcohol, dietary habits and physical activity) and environmental factors (e.g. overcrowded housing and pollution) [11]. As indices of deprivation are constructed by combining variables that are also likely determinants of human longevity, the reported associations with LEB are thus not surprising. However, linear regression models used to quantify the association between LEB and deprivation should also acknowledge the imperfect nature of the latter by making suitable distributional assumptions on the residuals of the model. Accounting for spatial correlation is especially important so as to deliver reliable estimates of LEB. More specifically, ignoring spatial correlation can lead to unreliable standard errors on the regression coefficients that regulate the strength of the association between LEB and deprivation; see, for example, Thomson et al. [12] in the context of disease mapping using geostatistical methods. However, methodological challenges arise from the fact that different Government agencies often release spatially aggregated information on LEB and other socio-demographic variables, including deprivation, at different spatial scales. For example, in the UK, the Life Events and Population Sources Division of the Office for National Statistics releases information on LEB by Middle Super Output Area (MSOA) while the index of multiple deprivation (IMD), published by the Ministry of Housing, Communities and Local Government, is available at a higher spatial resolution by Lower Super Output Area (LSOA). An example of this is given by Fig. 2 showing maps for male and female LEB and IMD in Liverpool, United Kingdom (UK). The rationale for calculating LEB at MSOA-level is that reliable estimates of LEB cannot be obtained from a population of less than 5000 individuals [13] and MSOAs satisfy this requirement, having 7200 inhabitants on average [14].

In the recent paper by [15], the authors investigate the association between LEB and IMD in England using a linear regression modelling framework. Their analysis is carried out at MSOA-level by taking the population-weighted average IMD based on the LSOAs falling in each of the corresponding MSOAs while assuming independent and identically distributed Gaussian residuals. This modelling approach ignores two important aspects: the within-MSOA variation which could result in a biased estimate for the regression coefficient associated with IMD; the residual spatial correlation in LEB, which affects the standard errors of the regression coefficient estimates [12]. Furthermore, the technique used by [15] can only be reliably applied when spatial units at different scales are nested within each other.

The issue of spatial misalignment has been widely addressed in the statistical literature; see [16, 17] for an overview. Our concern in this paper is with “areal-areal” misalignment, i.e. when data are available over misaligned, not necessarily nested, partitions of the same study area. A common approach used to address this problem is to predict the aggregated values of all the variables on a common set of spatial units and use the resulting predictions to build a regression model; [15] is an example of this [18] refers to this strategy as “krige and regress”. They show that the estimator of the regression coefficient is consistent but the variance estimator can be biased. More rigorous approaches have been developed by joint modelling of the outcome variable and the covariates. For example, [19] developed a joint model for outcomes observed at pixel-level and covariates at areal-level. The spatial correlation is modelled using conditionally autoregressive (CAR) models [20] for both pixel- and area-level spatial random effects. However, the use of CAR models for modelling outcomes aggregated over irregular spatial units (as in the case of LSOAs and MSOAs) is questionable because the adopted spatial structure is tied to the given partition of the study area, which is often drawn for administrative convenience. Also, [21] showed that when dealing with regions of varying size and shape, CAR models can induce counter-intuitive spatial correlation structure.

In this paper, our objectives are: (1) to develop a model-based geostatistical approach that allows the joint analysis of LEB and IMD data when these are available as spatially aggregated indices over misaligned partitions of the study area; (2) to carry out spatially continuous inference on LEB using spatially aggregated data. We illustrate our modelling approach through the analysis of LEB data from the Liverpool district council in the UK. Liverpool has been ranked as the most deprived local authority area in England in 2004, 2007 and 2010, and as the 4th most deprived in 2015 [22]. In 2018, LEB for both men and women was lower than the overall average in England [23]. Understanding the relationship between deprivation and life expectancy within a single conurbation helps to develop policies and evaluate interventions that are aimed at reducing health inequalities [24].

To address the aforementioned limitations of existing methods of inference, we develop a geostatistical framework that avoids the re-aggregation of IMD at MSOA-level. Instead, we jointly model LEB and IMD as aggregated outcomes of a spatially continuous stochastic process. More specifically, we model the spatial correlation across MSOAs for LEB and across LSOAs for IMD using inter-point distances based on a regular grid covering the whole of the study area. One of the main advantages of this approach is that it allows to carry out spatial prediction at any desired spatial scale, regardless of the format of the analysed data. The methodology presented in this paper can also be used to model any spatially aggregated health outcome and estimate its association with risk factors that may be available at a range of spatial scales.

All the analyses presented in this paper have been implemented in the R software environment (cran.r-project.org) and maps have been generated using the Q-GIS software (qgis.org). We provided the proof of the equations in Additional file 1. We provide the analysed data and the implemented R code in Additional files 2, 3, 4 and 5.

## Methods

### Data

#### **Index of multiple deprivation**

IMD is a measure of relative deprivation and can thus be used to rank neighbourhoods. It combines seven distinct domains of deprivation: income; employment; education; skills and training; health deprivation and disability; crime, barriers to housing and services; and living environment. Weighted cumulative models are used to compute the IMD score, with weights obtained via the maximum likelihood method for factor analysis [25, 26]. IMD data are made available either as a scores, deciles or ranks. In this study, we used the IMD score released in 2015, which was based on data collected between 2012 and 2013 and released by the UK Government.^1^ Larger values of the IMD score can be interpreted as corresponding to a higher level of deprivation of an area relative to the others [27].

#### Life expectancy at birth

Our outcome variable is the LEB released by the [28] (ONS). The ONS estimates LEB using life tables that are constructed by applying the Chiang method [29] to mortality data collected over five consecutive years, starting from 2009. This method assumes that the probability of dying is constant within a specified set of age intervals $a_{t-1}$ and $a_{t}$. The resulting estimator is

$$\begin{aligned} LEB = \sum _{t=1}^{T} \left[ (a_t - a_{t-1}) p_t + m_t d_t\right] \end{aligned}$$

where $p_t$ is the fraction of the total population that has not died in the time interval $(a_{t-1},a_t)$, $m_t$ is the average number of years lived in an interval by an individual who passes away in $(a_{t-1},a_t)$, $d_t$ is the fraction of the total population that dies in $(a_{t-1},a_t)$ between ages $a_{t-1}$ and $a_t$ and *T* is the number of age intervals. In our case, we have $T=19$, $(a_1,a_2)=(0,1)$, $(a_2, a_3)=(1,4)$ and for $t>3$, $a_t-a_{t-1} = 5$.

Life tables are usually constructed separately for males and females because of their different mortality patterns [30]. In the next section, we exploit the correlation between LEB for the two genders, and their association with IMD, in order to obtain more accurate estimates.

**Fig. 1:**
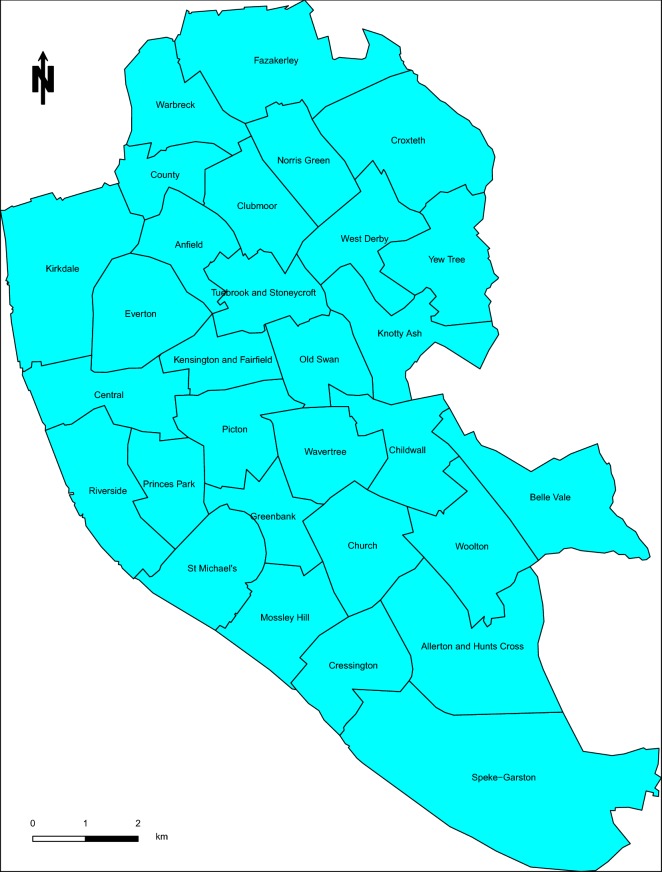
Map of Liverpool district council, UK showing the 30 electoral wards

Figure 1 shows the boundaries of the electoral wards (EWs) in Liverpool district and their names. In commenting the results, we shall refer to the different areas of the Liverpool district council based on the EWs in Fig. 1.

### Modelling framework

Let $LEB_{ij}$ denote the life expectancy at birth for males, if $i=1$, and females, if $i=2$, at the *j*-th MSOA, henceforth $MSOA_j$, for $j=1,\ldots ,n$. Similarly, we use $IMD_k$ to denote the IMD score for the *k*-th LSOA, henceforth $LSOA_k$, for $k = 1, \ldots , m$.

Define *U*(*x*) to be a spatially continuous Gaussian process, with stationary and isotropic exponential covariance function, i.e.

$$\begin{aligned} \mathrm{Cov}\{U(x), U(x')\} = \tau ^2 \exp \{-\Vert x-x'\Vert /\delta \}, \end{aligned}$$

where $\tau ^2$ is the variance and $\delta$ is a scale parameter regulating the rate of decay of the spatial correlation for increasing Euclidean distance $\Vert x-x'\Vert$ between any two locations *x* and $x'$.

We then model the cross-correlation in space between LEB and IMD through *U*(*x*) as follows. Define the averaged spatial processes based on *U*(*x*) over LSOAs and MSOAs as $U_j = \int _{MSOA_j} U(x) \, dx/|MSOA_j|$ and $U_k^* = \int _{LSOA_k} U(x) \, dx/|LSOA_k|$, where $|\mathcal {A}|$ corresponds to the area in m$^2$ of a spatial unit $\mathcal {A}$. The proposed joint model for $LEB_{ij}$ and $IMD_k$ takes the form

1 $$\begin{aligned} {\left\{ \begin{array}{ll} LEB_{ij} = \alpha _{i} + \beta _{i}U_{j} + T_{ij} &{} \text{ for } i=1,2;j=1,\ldots ,n \\ IMD_{k} = \gamma + U^*_{k} + V_{k} &{} \text{ for } k=1,\ldots ,m \end{array}\right. }, \end{aligned}$$

where the $\beta _{i}$ parameters quantify the strength of the association between LEB and IMD, whilst the $\alpha _i$ and $\gamma$ are intercept parameters. Also in (1), the $V_k$ are i.i.d. Gaussian variables with mean zero and variance $\nu ^2$, whilst ($T_{1j}$, $T_{2j}$) are i.i.d. bivariate Gaussian variables with mean zero and covariance matrix

$$\begin{aligned} \Omega =\left( \begin{matrix} \omega _{1}^2 &{} \omega _{12} \\ \omega _{12} &{} \omega _{2}^2 \end{matrix} \right) . \end{aligned}$$

It follows that the covariance between $LEB_{ij}$ and $IMD_{k}$ is

2 $$\begin{aligned} \mathrm{Cov}\{LEB_{ij}, IMD_{k}\} = \frac{\beta _{i}\tau ^2}{|MSOA_{j}||LSOA_{k}|} f(MSOA_{j},LSOA_{k}; \delta ), \end{aligned}$$

where

3 $$\begin{aligned} & f(MSOA_{j},LSOA_{k}; \delta ) \\ & = \int _{MSOA_{j}}\int _{LSOA_{k}} \exp \left\{ -\frac{\Vert x_j-x_{k}\Vert }{\delta }\right\} d x_j \, d x_{k}. \end{aligned}$$

In order to understand how much of the spatial variation in LEB is explained by IMD, we compare the fitted model (1) with the special case of no association with IMD, i.e. $\beta _1=\beta _2=0$.

An important feature of the spatial covariance structure defined by Eq. (2) is that it accounts for the different shapes and sizes of the various areal units involved.

### Inference: parameter estimation and spatially continuous prediction

Let $LEB_i = (LEB_{i1}, \ldots , LEB_{in})$ and $IMD = (IMD_{1}, \ldots , IMD_{m})$ and denote by $\theta$ the vector of model parameters. Also, let $\Sigma _{LSOA}$ and $\Sigma _{MSOA}$ be the spatial covariance matrices of the IMD at LSOA- and MSOA-level, respectively. The $(k,k')$ entry for $\Sigma _{LSOA}$ is

4 $$\begin{aligned} (\Sigma _{LSOA})_{kk'} = \frac{\tau ^2}{|LSOA_{k}||LSOA_{k'}|} f(LSOA_{k},LSOA_{k'}; \delta ) \end{aligned}$$

where $f(LSOA_{k},LSOA_{k'}; \delta )$ is as specified in Eq. (3). The elements of $\Sigma _{MSOA}$ are obtained similarly, replacing the domains of the integrals that define (4) with those of the corresponding MSOAs. Using $[\cdot ]$ as a shorthand notation for “the density function of the random variable $\cdot$,” the likelihood function for $\theta$ can now be expressed as

5 $$\begin{aligned} L(\theta )=\, & {} [LEB_1, LEB_2, IMD ; \theta ] \nonumber \\=\, & {} [LEB_1, LEB_2 \, | \, IMD ; \theta ] [IMD; \theta ] , \end{aligned}$$

where $[IMD; \theta ]$ is multivariate Gaussian with mean $\gamma \mathbb {1}_{m \times 1}$ and covariance $\Sigma _{LSOA} + \nu ^2 \mathbb {I}_m.$ Finally, $[LEB_1, LEB_2 \, | \, IMD ; \theta ]$ is a multivariate Gaussian with mean

6 $$\begin{aligned} \alpha \oplus \mathbb {1}_{n \times 1} + C^\top \Sigma _{LSOA}^{-1} (IMD - \gamma \mathbb {1}_{m \times 1}), \end{aligned}$$

and covariance

7 $$\begin{aligned} \Sigma _{LEB} - C^\top \Sigma _{LSOA}^{-1} C, \end{aligned}$$

where: $\alpha = (\alpha _1, \alpha _2)^\top$; $\oplus$ is the Kronecker product; $C = (C_1, C_2)^\top$ with $C_i$ being the cross-covariance between $LEB_{i}$ and *IMD* whose entries are given by Eq. (2); finally,

$$\begin{aligned} \Sigma _{LEB} = \begin{pmatrix} \beta _1^2 \Sigma _{MSOA} + w_1^2 \mathbb {I}_{n} &{} \beta _1 \beta _2 \Sigma _{MSOA} + w_{12} \mathbb {I}_n \\ \beta _1 \beta _2 \Sigma _{MSOA} + w_{12} \mathbb {I}_{n} &{} \beta _2^2 \Sigma _{MSOA} + w_2^2 \mathbb {I}_{n} \end{pmatrix}. \end{aligned}$$

We calculate each of the integrals in (2) and (4) using the numerical approximation described in Section 3 of [31]. Finally, we estimate $\theta$ through maximization of the likelihood function in (5).

To quantify the contribution of IMD in explaining the spatial variation in LEB, we use the fraction of the total variance explained, given by

8 $$\begin{aligned} \frac{\mathrm{Var}\{\beta _{i} U_{j}\}}{\mathrm{Var}\{LEB_{ij}\}} = \frac{\beta _{i}^2\tau ^2}{\beta _{i}^2\tau ^2 + \omega _{i}^2}, \end{aligned}$$

with $i=1$ for the male population and $i=2$ for the females, respectively.

We carry out spatial prediction over a regular grid at a spatial resolution of 250 by 250 m, covering the whole of the Liverpool council area. Let $\{x_{1},\ldots ,x_{q}\}$ be the set of points forming the grid, with $q=1787$, and let $LEB_{i}(x_{h}) = \alpha _{i} + \beta _{i}U(x_{h})$ be the unobserved value of LEB at $x_{h}$, for $h=1,\dots ,q$. Now, write $LEB^* = (LEB_1(x_1), \ldots , LEB_1(x_q), LEB_2(x_1), \ldots , LEB_2(x_q))^\top$; the predictive distribution for $LEB^*$, i.e. its conditional distribution given the data, is multivariate Gaussian with mean

9 $$\begin{aligned} \alpha \oplus \mathbb {1}_{q \times 1} + D^\top \Sigma _{LEB}^{-1} (LEB - \alpha \oplus \mathbb {1}_{n \times 1}), \end{aligned}$$

and covariance matrix

10 $$\begin{aligned} \Sigma _{LEB^*} - D^\top \Sigma _{LEB}^{-1} D. \end{aligned}$$

In (10), the $(h,h')$-th element of $\Sigma _{LEB^*}$ is given by $(\Sigma _{LEB^*})_{hh'} = \tau ^2 \exp \{-\Vert x_{h}-x_{h'}\Vert /\delta \}$. Also,

$$\begin{aligned} D= \left( \begin{matrix} D_{1} \\ D_{2} \end{matrix} \right) \end{aligned}$$

where $D_{i}$ is the $n \times q$ matrix whose *h*-th column is $(d_{1}(x_h), \ldots , d_{n}(x_h))$, and $d_{j}(x_h) = \beta _{i}^2 \tau ^2 \int _{MSOA_j} \exp \left\{ -\Vert x_h-x\Vert /\delta \right\} \, dx$.

Using the above results, we can then draw samples for $LEB^*$ and obtain any predictive summary of interest. For example, to identify areas in the Liverpool council district that are highly likely to fall below a threshold *l*, we map the non-exceedance probabilities (NEPs)

11 $$\begin{aligned} NEP_{i}(x) = Pr(LEB_{i}(x) < l \, | \, LEB_{1}, LEB_{2}, IMD). \end{aligned}$$

In the results shown in the next section, we set *l* to be England-wide average years for males ($l=79.2$ years) and females ($l=82.9$ years). Values of NEP close to 1 indicate that LEB is highly likely to lie below *l*. Conversely, values close to 0 indicate locations whose LEB is highly likely to be above *l*. Finally, locations with values around 0.5 are equally likely to be below or above *l*, thus corresponding to the scenario with highest uncertainty.

Our results have been made publicly available at the following link http://fhm-chicas-apps.lancs.ac.uk/shiny/users/johnsono/LEBLiverpool/, where interactive maps for NEPs can be generated from our model for any chosen threshold *l*.

### Model validation: testing for residual spatial correlation

One of the main assumptions of the fitted bivariate model (1) is that all the spatial variation in LEB is captured by the IMD. To validate this assumption, we proceed as follows. We first estimate the $T_{ij}$ as

$$\begin{aligned} LEB_{ij} - \hat{\alpha }_{i}-\hat{\beta }_{i}\hat{U}_{j} \quad \text { for }i=1,2; j=1,\ldots ,n \end{aligned}$$

where $\hat{\alpha }_{i}$ and $\hat{\beta }_{i}$ are the maximum likelihood estimates and $\hat{U}_{j}$ is the predictive mean of $U_{j}$. For each MSOA, we then extract the centroid associated with each of the $\hat{T}_{ij}$. For both males ($i=1$) and females ($i=2$), we then compute the empirical variogram given by

12 $$\begin{aligned} \hat{\gamma }_i(\mathcal {U}) = \frac{1}{2|\mathcal {U}|} \sum _{(j,k) \in \mathcal {U}} ( \hat{T}_{ij} - \hat{T}_{i'j} )^2, \end{aligned}$$

where $\mathcal {U} = [u_0, u_1]$ is the set of all pairs of all pairs of centroids that no less than $u_0$ and no more than $u_1$ distant apart, and $|\mathcal {U} |$ is the number of pairs within the set. In the current analysis, we construct the empirical variogram by segmenting the interval [0, 10] (km) into 12 equally spaced intervals.

In order to test whether the observed $\hat{\gamma }_i(\mathcal {U})$ is compatible with assumption of no residual spatial correlation, we use the following Monte Carlo approach to construct 95% tolerance intervals around $\hat{\gamma }_i(\mathcal {U})$:

1. Permute the order of $T_ij$, while holding the centroid of the MSOAs fixed;
2. Compute the empirical variogram $\hat{\gamma }_i(\mathcal {U})$ for the permuted $T_{ij}$;
3. Repeat step 1 and 2 for a large number of times, say *B*;
4. Use the resulting *B* empirical variograms to generate 95% tolerance intervals at each of the predefined distance bins.

If $\hat{\gamma }_i(\mathcal {U})$ lies within the 95$\%$ tolerance intervals, we conclude that the assumption that the IMD fully captures the spatial variation in LEB is supported by the data. If, instead, $\hat{\gamma }_i(\mathcal {U})$ falls outside the 95$\%$ tolerance intervals, we conclude that the data show evidence against the fitted model in (1).

### Assessment of the coverage probabilities for the regression parameters and the spatial predictions

In this section, we outline a simulation study which we carry out in order to assess the reliability of the confidence intervals generated for the regression coefficients $\beta _{i}$, the spatially continuous predictions and the MSOA-level predictions for LEB. This is especially important in our case as we carry out spatial predictions by plugging-in the maximum likelihood estimates, hence ignoring parameter uncertainty.

We then simulate $B=10,000$ data sets under the bivariate the model in (1) using the administrative boundaries of Liverpool and proceed through the following iterative steps:

1. Simulate the spatially continuous process *U*(*x*) over a 150 $\times$ 150 metres grid.
2. Simulate the spatially continuous surface for IMD and LEB on the same regular grid.
3. Average the LEB over the MSOAs boundaries and the IMD over the LSOAs boundaries.
4. Fit the model in (1) and compute confidence intervals of coverage $\alpha$ for $\beta _1$ and $\beta _2$.
5. Compute the prediction intervals of coverage $\alpha$ for the LEB at MSOA-level and over the 150 $\times$ 150 metres grid.

In this simulation we set the true value of the parameters to the point estimate reported for Model 1 in Table 1. We let the coverage probability $\alpha$ vary over the set $\{5i/100: i=1,2,\ldots ,19\}$. Using the resulting 10,000 confidence intervals in step 4 and prediction intervals in step 5, we compute the fraction of times that the true values fall within those intervals in order to obtain the actual coverage.

**Table 1:** Point estimates and 95$\%$ confidence intervals (CI) for the three model parameters

Parameter	Model 1		Model2	
	Estimate	CI 95%	Estimate	CI 95%
$\alpha _1$	75.466	(75.596, 76.135)	75.131	(74.990, 75.272)
$\alpha _2$	81.120	(80.883, 81.357)	81.375	(80.927, 81.823)
$\beta _1$	− 0.154	(− 0.180, −0.128)	–	–
$\beta _2$	− 0.129	(− 0.167, −0.091)	–	–
$\log \omega ^2_1$	1.810	(1.494, 2.126)	3.036	(2.955, 3.117)
$\log \omega ^2_2$	2.581	(2.272, 2.890)	3.160	(3.033, 3.287)
$\log \omega _{12}$	1.671	(1.257, 2.086)	2.871	(2.768, 2.974)
$\gamma$	39.221	(28.242, 50.200)	39.190	(28.073, 50.306)
$\log \tau ^2$	6.226	(3.611, 8.841)	6.232	(5.678, 6.586)
$\log \delta$	7.336	(6.845, 7.827)	7.349	(6.318, 7.846)
$\log \nu ^2$	2.586	(2.244, 2.927)	2.589	(2.064, 2.932)
Log-likelihood	− 1429.491		− 1465.432	

## Results

Table 1 shows the point and interval estimates for the model with (Model 1) and without (Model 2) IMD. The likelihood-ratio test for the null hypothesis $\beta _{1}=\beta _{2}=0$ yields a p-value smaller than 0.001, hence indicating that Model 1 is a better fit to data. We find that the fraction of total variance explained (see Eq. 8)) is about 38.92% for females and 63.52% for males, respectively. We estimate that the range of the spatial correlation, defined as the distance beyond which the correlation is below 0.05, is approximately 4.6 km. The correlation in LEB between males and females, given by ratio $\omega _{12}/(\omega _{1} \omega _{2})$, is 0.59 with associated 95$\%$ confidence interval (0.31, 0.90).

**Fig. 2:**
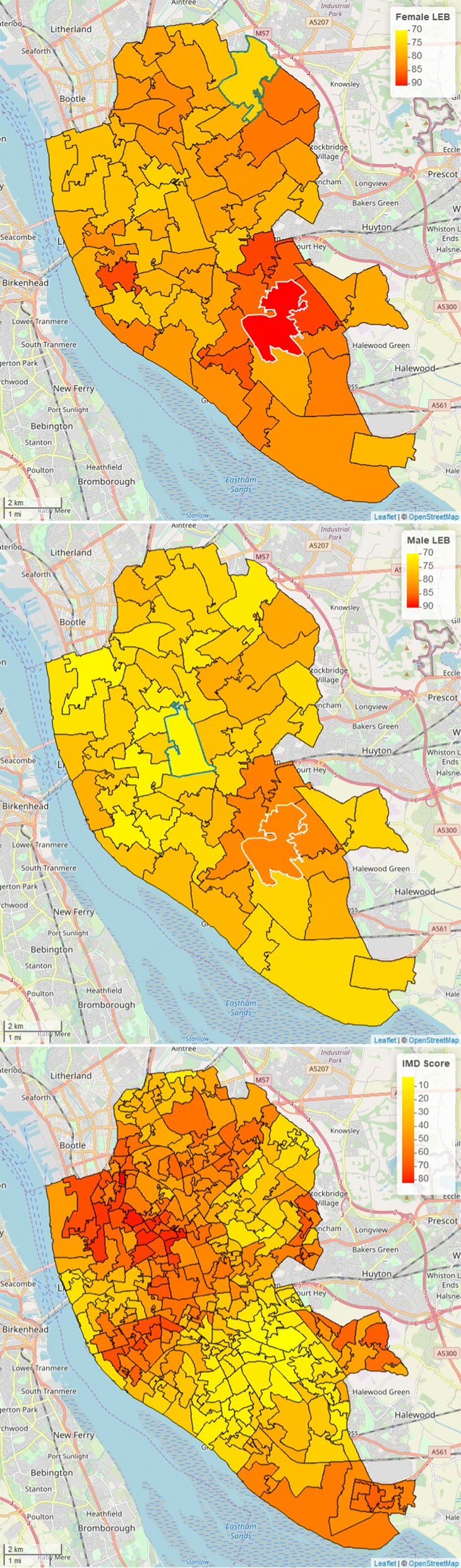
Maps of the estimated female (upper panel) and male (middle panel) life expectancy at birth (LEB) and index of multiple deprivation (IMD) (lower panel). Middle Super Output Area (MSOA) with boundaries coloured in green correspond to the lowest estimated LEB, whilst those in white to the highest. For males, the lowest estimated LEB is 70.2 years and the highest is 85.2 years; for females, the lowest is 73.5 years and the highest is 89.6 years

**Fig. 3:**
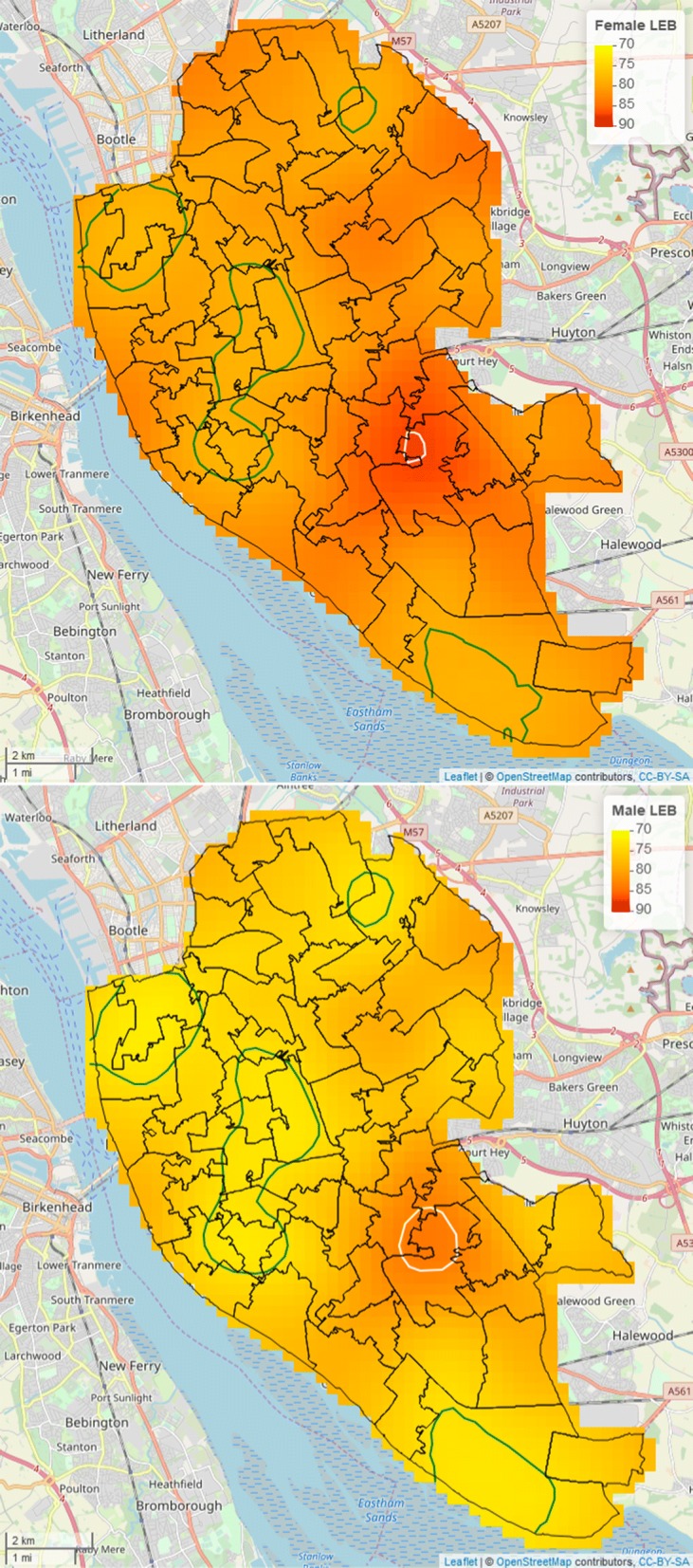
Spatially continuous prediction maps of female (upper panel) and male (lower panel) life expectancy at birth (LEB) in Liverpool, UK. In the upper panel, the white contour lines are for a LEB of 89.6 years and the green contour lines for a LEB of 73.5 yers; in the lower panel, the white contour lines correspond to 70.2 years and the green contour lines to 85.2 years

**Fig. 4:**
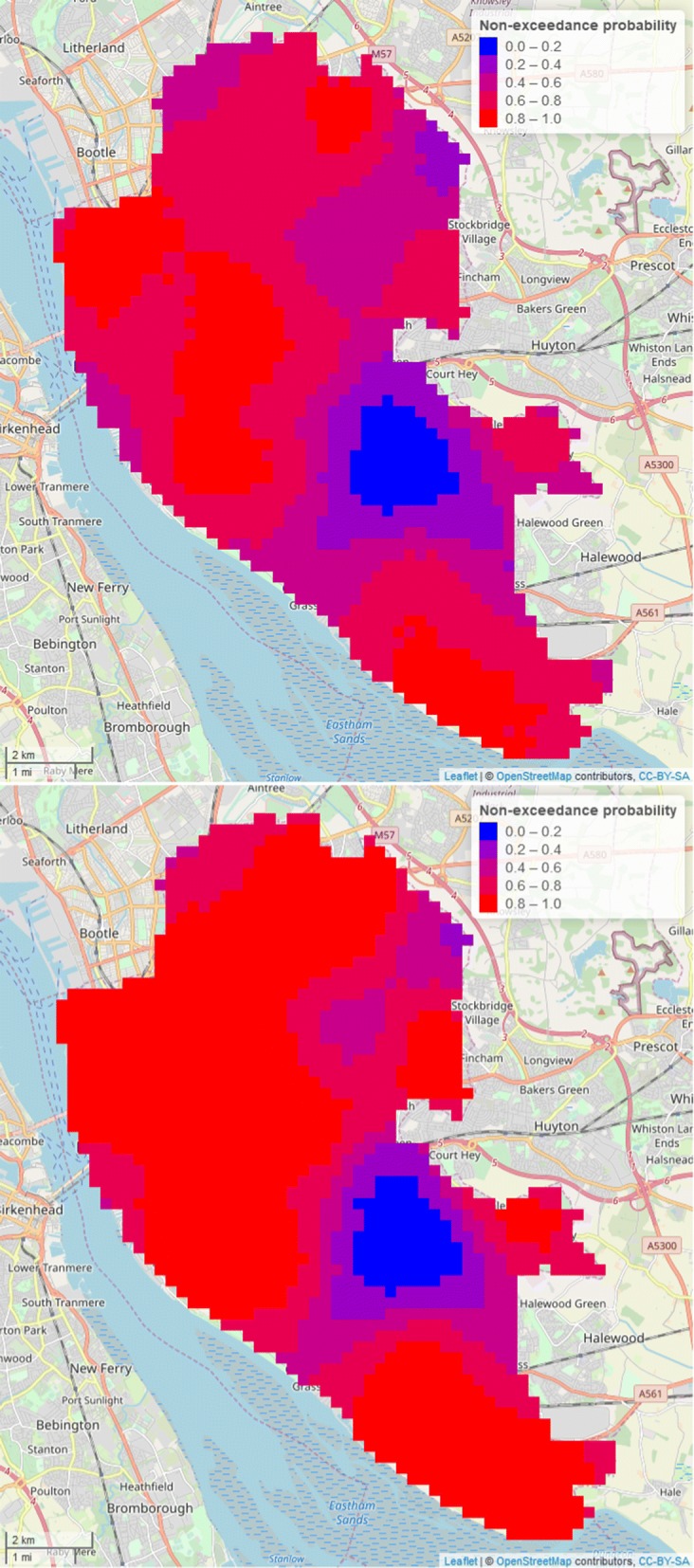
Maps of the non-exceedance probability of female (upper panel) and male (lower panel) life expectancy at birth (LEB), with threshold 82.9 and 79.2 (average LEB in England, UK), respectively in Liverpool, UK

Figure 2 (upper and middle panel) shows the estimated surface of LEB at MSOA-level for females and males. As expected, female LEB is consistently higher than that for males, as also reflected in the spatially continuous predictions of Fig. 3. In contrasting the maps of Fig. 2 with those of Fig. 3, we notice that spatially continuous predictions provide useful insights into the variation in LEB within MSOAs that is otherwise hidden by the aggregated estimates at MSOA-level. To demonstrate this, we selected the MSOA with the lowest and largest estimated value in LEB for both males and females; these MSOAs are identified identified by the white (largest LEB) and green (lowest LEB) boundaries in upper and middle panels of Fig. 2. More specifically, for males, the lowest estimated value in LEB at MSOA-level is about 70.2 years and the largest is 85.2 years, whilst for females these are respectively 73.5 years and 89.6 years. In the maps of Fig. 4, we then draw the contour lines for these same values in LEB. These reveal the actual extent of the areas where LEB reaches its highest and lowest values, that cannot be possibly discerned from Fig. 2: the white contour lines encompass a relatively small at the intersection of Childwall, Woolton and Church; the green contour lines, instead, delineate a wide area consisting of three disjoint sub-regions in the north-west and north-east of Liverpool.

Figure 4 shows the non-exceedance probability maps of female and male LEB, with thresholds of 82.9 years and 79.2 years, respectively. These two values also correspond to the national average LEB in England for the two genders. For females, we find that LEB is at least 80$\%$ likely to be below 82.9 years in the areas of Kirkdale, Kensington and Fairfield and Princes Park; for males, a wider area is instead identified, comprising those same EWs with the addition of Fazakerley, Norris Green, Clubmoor, County, Anfield, Everton, Tuebrook and Stoneycroft, Picton, Central, St Michaels and Speke-Garston. On the other hand, areas that are at least 80$\%$ to be above the England-wide averages are are found in the EWs of Childwall, Woolton and Church for both males and females. In the EWs of West Derby and Mossley Hill the model is most uncertain as these are equally likely to have a LEB above or below the chosen thresholds for the both males and females.

**Fig. 5:**
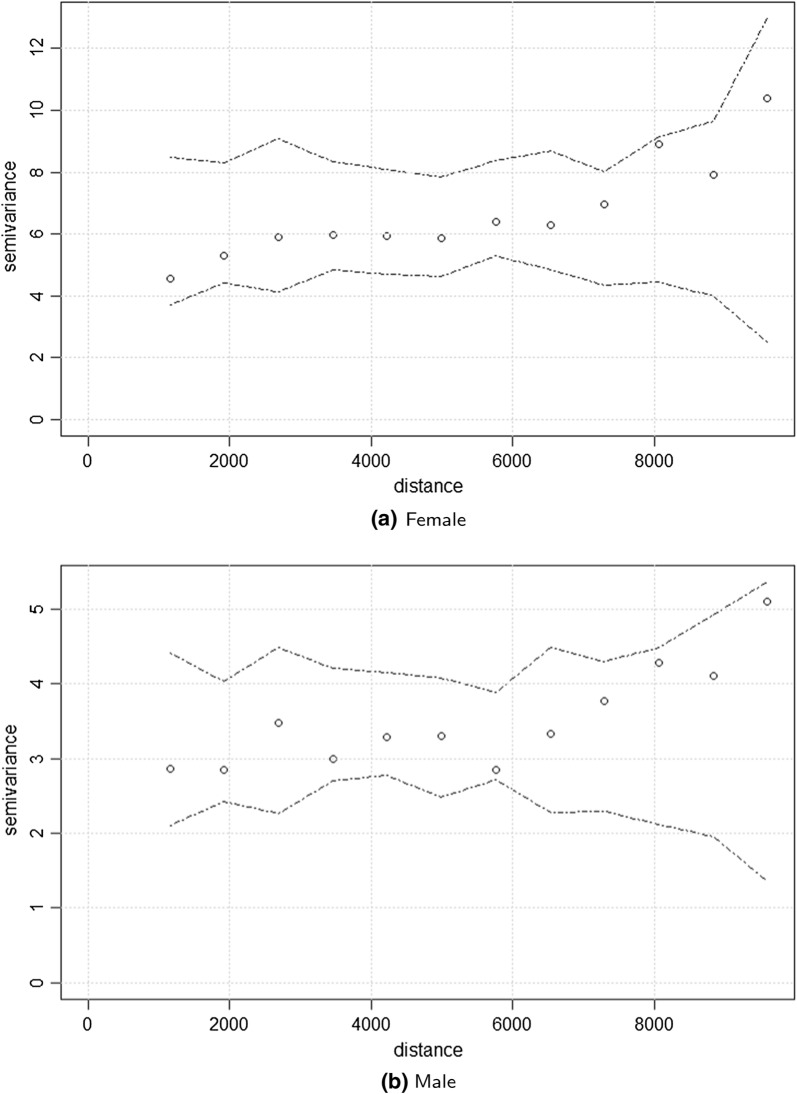
Plots of the observed variograms (points) and the 95% tolerance bandwidth (dashed lines) generated under the assumption of absence of residual spatial correlation

Figure 5 the results for the variogram-based validation procedure. Since the observed variograms for both males and females lie within the 95% band, we interpret this as evidence that the data do not show any additional residual spatial correlation. This leads us to conclude that the IMD was able to explain most of the spatial variation in LEB.

**Fig. 6:**
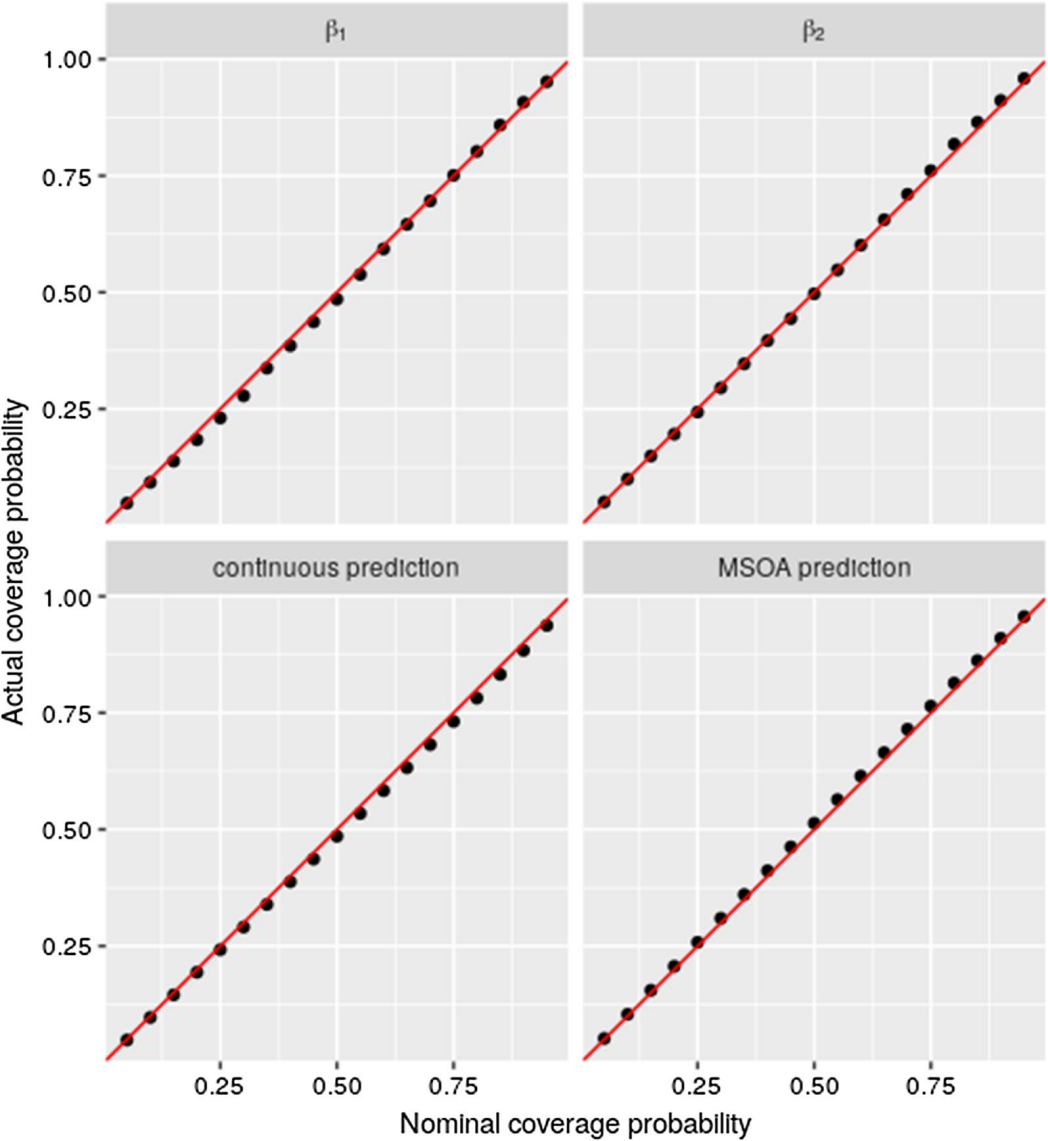
Scatter plots of the actual against the nominal coverage for the confidence intervals generated for $\beta _{1}$ and $\beta _{2}$ (upper panels), and for the spatially continuous and MSOA-level predictions of LEB (lower panels). The red lines in each panel correspond to the identity line

Figure 6 shows the scatter plots of the actual coverage, obtained from the simulation study, against the nominal coverage. For the spatial predictions, the actual coverage is averaged over all the MSOAs and over the regular grid, respectively. The plots show a strong concordance between actual and nominal coverage levels. We then conclude that the interval estimates for the regression coefficients and the spatial predictions generated by the fitted model are in fact reliable when using plug-in estimates.

## Discussion

We have developed a model-based geostatistical approach that allows to model the relationship between life expectancy and the index of multiple deprivation when these are provided over misaligned partitions of the study area. Unlike existing methods of analysis (e.g. [15]), one of the main advantages of our approach is that it allows to combine information from multiple data sources without coarsening their resolution to a common spatial scale. The underpinning principle of our modelling framework is that spatially aggregated data should be treated as the realization of an aggregated spatially continuous stochastic process. This approach is strongly linked to that of [32] who propose the use of an integrated log-Gaussian Cox process to model disease counts at areal-level. As result of this, the proposed modelling paradigm allows to carry out spatially continuous inference which would be otherwise infeasible if the spatial models were tied to the specific data-format at which LEB and IMD are provided. Conditionally autoregressive models [20] are one of the most commonly used approaches to analyse areal-level data that suffer from this limitation [19, 33].

Our novel methodology has highlighted the importance of dealing with variation in LEB occurring within areal units. In our application, the use of spatially continuous predictions was especially useful in order to visualize patterns in LEB that were hidden by the aggregated estimates. Furthermore, the use of non-exceedance probabilities also provides a way of measuring uncertainty in relation to a predefined threshold in LEB in order to identify areas that need urgent intervention.

One of the limitations of the model defined by Eq. (1), is that all the spatial variation in LEB and IMD is modelled through a single spatial process *U*(*x*). The model could then be made more flexible through the introduction of a second spatial process, say *W*(*x*), into the first line of Eq. (1), i.e.

$$\begin{aligned} LEB_{ij} = \alpha _i + \beta _i U_{j} + W_{j} + T_{ij}, \quad \text { for } i=1,2; j=1,\ldots ,n \end{aligned}$$

where $W_{j} = |MSOA_{j}|^{-1}\int _{MSOA_{j}} W(x) \, dx$. In this model, the $W_{j}$ would allow to account for unexplained spatial variation in LEB that is unrelated to IMD. However, in our attempt to fit such a model, we incurred in identifiability issues as the estimated spatial scale for the process *W*(*x*) was well below the extent of the smallest MSOA. This also suggests that most of the large scale spatial variation in LEB is in fact well captured by the IMD and that unexplained variation occurring on a smaller spatial scale is instead accounted for by the unstructured component of the model $T_{ij}$.

Although our application to mapping LEB in Liverpool only dealt with areal misalignment, our methodology is more widely applicable to almost any scenarios of spatial misalignment. Consider, for example, the case where a second spatially varying factor associated with LEB is available in raster format over a regular grid, say $\{\tilde{x}_1,\ldots ,\tilde{x}_q\}$, covering the whole of the Liverpool council area. Let $V(\tilde{x}_k)$ denote the value of such a variable at the grid location $\tilde{x}_k$, for $k=1,\ldots ,q$. Model (1) could then be extended by replacing the first line with

$$\begin{aligned} LEB_{ij} = \alpha _i + \beta _i U_j + \delta _i V_j + T_{ij}, \end{aligned}$$

where $V_{j} = |MSOA_{j}|^{-1}\int _{MSOA_{j}} V(x) \, ~ dx$. Assuming a high enough spatial resolution of the raster file for *V*(*x*), this integral could then be approximated by taking a sample average over the grid locations falling within $MSOA_{j}$. If, instead, the grid is too coarse, spatial variation in *V*(*x*) within pixels can be accounted for by building a geostatistical model in a similar fashion as for the IMD in the second line of Eq. (1).

## Conclusion

We have developed a novel joint geostatsitical approach to model the relationship between life expectancy at birth and the index of multiple deprivation while dealing with the issue of spatial misalignment. Unlike existing spatial methods based on conditional autoregressive models, one of the main strengths of the proposed modelling framework is the ability to carry out spatially continuous predictions regardless of the format of the data. Furthermore, it is also more widely applicable to more complex data scenarios where information is provided at a range of spatial scales, from pixel-level to areal-level.

## Supplementary information

**Additional file 1.** The proof of the Eqs. (2)–(10).

**Additional file 2.** The R script to reproduce the analysis.

**Additional file 3.** The life expectancy at birth data.

**Additional file 4.** The index of multiple deprivation data.

**Additional file 5.** The readme file that provides that guides on how to reproduce the anaylysis.

## Data Availability

All data analysed in the paper and associated R code is available as addiitonal files.

## Abbreviations

LEB: Life expectancy at birth
IMD: Index of multiple deprivation
LSOA: Lower Super Output Area
MSOA: Middle Super Output Area
UK: United Kingdom

## Footnotes

1 https://www.gov.uk/government/statistics/english-indices-of-deprivation-2015.
