# Supplementary material for: Dealing with spatial misalignment to model the relationship between deprivation and life expectancy: a model-based geostatistical approach
Source: Int J Health Geogr. 2020 Mar 4;19:6. doi: 10.1186/s12942-020-00200-w (PMC7057663; doi:10.1186/s12942-020-00200-w)
Supplement: Supplementary file 1 — Additional file 1. The proof of the Eqs. (2)–(10). [file 12942_2020_200_MOESM1_ESM.pdf]

Additional file 1: Dealing with spatial misalignment to model the relationship between deprivation and life expectancy: A model-based geostatistical approach

Olatunji Johnson, Peter Diggle, Emanuele Giorgi

February 27, 2020

Appendix

A Derivation of the equation

Let LEB_{ij} denote the life expectancy at birth for males, if $i = 1$, and females, if $i = 2$, at the j-th MSOA, henceforth $MSOA_j$, for $j = 1, \dots, n$. Similarly, we use IMD_k to denote the IMD score for the k-th LSOA, henceforth $LSOA_k$, for $k = 1, \dots, m$.

Define $U(x)$ to be a spatially continuous Gaussian process, with stationary and isotropic exponential covariance function, i.e.

$$\text{Cov}\{U(x), U(x')\} = \tau^2 \exp\{-\|x - x'\|/\delta\},$$

where τ^2 is the variance and δ is a scale parameter regulating the rate of decay of the spatial correlation for increasing Euclidean distance $\|x - x'\|$ between any two locations x and x'.

We then model the cross-correlation in space between LEB and IMD through $U(x)$ as follows. Define the averaged spatial processes based on $U(x)$ over LSOAs and MSOAs as $U_j = \int_{MSOA_j} U(x)dx/|MSOA_j|$ and $U_k^* = \int_{LSOA_k} U(x)dx/|LSOA_k|$, where $|\mathcal{A}|$ corresponds to the area in m^2 of a spatial unit $\mathcal{A}$. The proposed joint model for LEB_{ij} and IMD_k takes the form

$$\begin{cases} LEB_{ij} = \alpha_i + \beta_i U_j + T_{ij} & \text{for } i = 1, 2; j = 1, \dots, n \\ IMD_k = \gamma + U_k^* + V_k & \text{for } k = 1, \dots, m \end{cases}, \quad (1)$$

where the β_i parameters quantify the strength of the association between LEB and IMD, whilst the α_i and γ are intercept parameters. Also in (1), the V_k are i.i.d. Gaussian variables with mean zero and variance ν^2, whilst (T_{1j}, T_{2j})

are i.i.d. bivariate Gaussian variables with mean zero and covariance matrix

$$\Omega = \begin{pmatrix} \omega_1^2 & \omega_{12} \\ \omega_{12} & \omega_2^2 \end{pmatrix}.$$

Theorem A.1.

$$\mathrm{Cov}\{LEB_{ij}, IMD_k\} = \frac{\beta_i \tau^2}{|MSOA_j||LSOA_k|} f(MSOA_j, LSOA_k; \delta), \qquad (2)$$

where

$$f(MSOA_j, LSOA_k; \delta) = \int_{MSOA_j} \int_{LSOA_k} \exp\left\{-\frac{\|x_j - x_k\|}{\delta}\right\} dx_j \, dx_k. \qquad (3)$$

Proof.

$$\begin{aligned}
\mathrm{Cov}\{LEB_{ij}, IMD_k\} &= \mathrm{Cov}\{\alpha_i + \beta_i U_j + T_{ij}, \gamma + U_k^* + V_k\} \\
&= \mathrm{Cov}\{\alpha_i, \gamma\} + \mathrm{Cov}\{\alpha_i, U_k^*\} + \mathrm{Cov}\{\alpha_i, V_k\} + \mathrm{Cov}\{\beta_i U_j, \gamma\} \\
&+ \mathrm{Cov}\{\beta_i U_j, U_k^*\} + \mathrm{Cov}\{\beta_i U_j, V_k\} + \mathrm{Cov}\{T_{ij}, \gamma\} \\
&+ \mathrm{Cov}\{T_{ij}, U_k^*\} + \mathrm{Cov}\{T_{ij}, V_k\} \\
&= \beta_i \mathrm{Cov}\{U_j, U_k^*\} \\
&= \beta_i \mathrm{Cov}\left\{\frac{1}{|MSOA_j|}\int_{MSOA_j} U(x_j)\, dx_j, \frac{1}{|LSOA_k|}\int_{LSOA_k} U(x_k)\, dx_k\right\} \\
&= \beta_i \frac{1}{|MSOA_j|}\int_{MSOA_j} \frac{1}{|LSOA_k|}\int_{LSOA_k} \mathrm{Cov}\left\{U(x_j), U(x_k)\right\} dx_j dx_k \\
&= \beta_i \frac{1}{|MSOA_j|}\frac{1}{|LSOA_k|}\int_{MSOA_j}\int_{LSOA_k} \tau^2 \exp\left\{-\frac{\|x_j - x_k\|}{\delta}\right\} dx_j dx_k \\
&= \frac{\beta_i \tau^2}{|MSOA_j||LSOA_k|}\int_{MSOA_j}\int_{LSOA_k} \exp\left\{-\frac{\|x_j - x_k\|}{\delta}\right\} dx_j dx_k \\
&= \frac{\beta_i \tau^2}{|MSOA_j||LSOA_k|} f(MSOA_j, LSOA_k; \delta),
\end{aligned}$$

where $\mathrm{Cov}\{\alpha_i, \gamma\} = 0$, $\mathrm{Cov}\{\alpha_i, U_k^*\} = 0$, $\mathrm{Cov}\{\alpha_i, V_k\}$, $\mathrm{Cov}\{\beta_i U_j, \gamma\} = 0$, $\mathrm{Cov}\{\beta_i U_j, V_k\} = 0$, $\mathrm{Cov}\{T_{ij}, \gamma\} = 0$, $\mathrm{Cov}\{T_{ij}, U_k^*\} = 0$, and $\mathrm{Cov}\{T_{ij}, V_k\} = 0$. □

Theorem A.2. *Let Σ_{LSOA} be the spatial covariance matrix of the IMD at LSOA-level. The (k, k') entry for Σ_{LSOA} is*

$$(\Sigma_{LSOA})_{kk'} = \frac{\tau^2}{|LSOA_k||LSOA_{k'}|} f(LSOA_k, LSOA_{k'}; \delta) \qquad (4)$$

Proof. The (k, k') entry for Σ_{LSOA} is

$$
\begin{aligned}
\text{Cov}\{IMD_k, IMD_{k'}\} &= \text{Cov}\{\gamma + U_k^* + V_k, \gamma + U_{k'}^* + V_{k'}\} \\
&= \text{Cov}\{\gamma, \gamma\} + \text{Cov}\{\gamma, U_{k'}^*\} + \text{Cov}\{\gamma, V_{k'}\} + \text{Cov}\{U_k^*, \gamma\} \\
&+ \text{Cov}\{U_k^*, U_{k'}^*\} + \text{Cov}\{U_k^*, V_{k'}\} + \text{Cov}\{V_k, \gamma\} \\
&+ \text{Cov}\{V_k, U_{k'}^*\} + \text{Cov}\{V_k, V_{k'}\} \\
&= \text{Cov}\{U_k^*, U_{k'}^*\} \\
&= \text{Cov}\left\{\frac{1}{|LSOA_k|}\int_{LSOA_k} U(x_k)\, dx_k, \frac{1}{|LSOA_k'|}\int_{LSOA_k'} U(x_k')\, dx_k'\right\} \\
&= \frac{1}{|LSOA_k|}\int_{LSOA_k} \frac{1}{|LSOA_k'|}\int_{LSOA_k'} \text{Cov}\left\{U(x_k), U(x_k')\right\}\, dx_k dx_k' \\
&= \frac{1}{|LSOA_k|}\frac{1}{|LSOA_k'|}\int_{LSOA_k}\int_{LSOA_k'} \tau^2 \exp\left\{-\frac{\|x_k - x_{k'}\|}{\delta}\right\}\, dx_k dx_k' \\
&= \frac{\tau^2}{|LSOA_k||LSOA_{k'}|}\int_{LSOA_k}\int_{LSOA_k'} \exp\left\{-\frac{\|x_k - x_{k'}\|}{\delta}\right\}\, dx_k dx_k' \\
&= \frac{\tau^2}{|LSOA_k||LSOA_{k'}|} f(LSOA_k, LSOA_{k'}; \delta),
\end{aligned}
$$

where $\text{Cov}\{\gamma, \gamma\} = 0$, $\text{Cov}\{\gamma, U_{k'}^*\} = 0$, $\text{Cov}\{\gamma, V_{k'}\}$, $\text{Cov}\{U_k^*, \gamma\} = 0$, $\text{Cov}\{U_k^*, V_{k'}\} = 0$, $\text{Cov}\{V_k, \gamma\} = 0$, $\text{Cov}\{V_k, U_{k'}^*\} = 0$, and $\text{Cov}\{V_k, V_{k'}\} = 0$. □

Lemma A.3. *Suppose a multivariate Guassian random vector X is partitioned into two component $X = (X_1, X_2)^T$, where X_1 has q_1 components and X_2 has q_2 components. Then the joint distribution of X_1 and X_2 has mean vector*

$$X = \begin{bmatrix} X_1 \\ X_2 \end{bmatrix} \sim \mathcal{N}\left(\begin{bmatrix} \mu_1 \\ \mu_2 \end{bmatrix}, \begin{bmatrix} \Sigma_{11} & \Sigma_{12} \\ \Sigma_{21} & \Sigma_{22} \end{bmatrix}\right),$$

where μ_i has length $q_i : i = 1, 2$ and Σ_{ij} is a $q_i \times q_j$ matrix for $i, j = 1, 2$. Then the conditional distribution of X_1 given $X_2 = x_2$ follows a Gaussian distribution with mean

$$\mathbb{E}[X_1|X_2] = \mu_1 + \Sigma_{12}\Sigma_{22}^{-1}(x_2 - \mu_2)$$

and variance

$$\mathbb{V}[X_1|X_2] = \Sigma_{11} - \Sigma_{12}\Sigma_{22}^{-1}\Sigma_{21}.$$

Theorem A.4. *$[LEB_1, LEB_2 \,|\, IMD; \theta]$ is a multivariate Gaussian with mean*

$$\alpha \oplus \mathbb{1}_{n\times 1} + C^\top \Sigma_{LSOA}^{-1}(IMD - \gamma \mathbb{1}_{m\times 1}), \tag{5}$$

and covariance

$$\Sigma_{LEB} - C^\top \Sigma_{LSOA}^{-1} C, \tag{6}$$

where: $\alpha = (\alpha_1, \alpha_2)^\top$; $\oplus$ is the Kronecker product; $C = (C_1, C_2)^\top$ with C_i being the cross-covariance between LEB_i and IMD whose entries are given by Equation (2); finally,

$$\Sigma_{LEB} = \begin{pmatrix} \beta_1^2 \Sigma_{MSOA} + w_1^2 \mathbb{I}_n & \beta_1\beta_2 \Sigma_{MSOA} + w_{12}\mathbb{I}_n \\ \beta_1\beta_2 \Sigma_{MSOA} + w_{12}\mathbb{I}_n & \beta_2^2 \Sigma_{MSOA} + w_2^2 \mathbb{I}_n \end{pmatrix}.$$

Proof. According to Lemma A.3, let $X = (LEB_1, LEB_2, IMD)^T$ be partitioned into two parts such that $X = ((LEB_1, LEB_2), IMD)^T$. Therefore, if the joint distribution of (LEB_1, LEB_2) and IMD is

$$X = \begin{bmatrix} (LEB_1, LEB_2) \\ IMD \end{bmatrix} \sim \mathcal{N}\left(\begin{bmatrix} \alpha \oplus \mathbb{1}_{n\times 1} \\ \gamma \mathbb{1}_{m\times 1} \end{bmatrix}, \begin{bmatrix} \Sigma_{LEB} & C^\top \\ C & \Sigma_{LSOA} \end{bmatrix} \right),$$

then the conditional distribution of (LEB_1, LEB_2) given IMD follows a Gaussian distribution with mean

$$\alpha \oplus \mathbb{1}_{n\times 1} + C^\top \Sigma_{LSOA}^{-1}(IMD - \gamma \mathbb{1}_{m\times 1}),$$

and covariance

$$\Sigma_{LEB} - C^\top \Sigma_{LSOA}^{-1} C,$$

where: $\alpha = (\alpha_1, \alpha_2)^\top$; $\oplus$ is the Kronecker product; $C = (C_1, C_2)^\top$ with C_i being the cross-covariance between LEB_i and IMD whose entries are given by Equation (2); finally,

$$\begin{aligned} \Sigma_{LEB} &= \text{Cov}\begin{bmatrix} LEB_1 \\ LEB_2 \end{bmatrix} \\ &= \begin{pmatrix} \text{Var}(LEB_1) & \text{Cov}(LEB_2, LEB_1) \\ \text{Cov}(LEB_1, LEB_2) & \text{Var}(LEB_2) \end{pmatrix} \\ &= \begin{pmatrix} \beta_1^2 \Sigma_{MSOA} + w_1^2 \mathbb{I}_n & \beta_1\beta_2 \Sigma_{MSOA} + w_{12}\mathbb{I}_n \\ \beta_1\beta_2 \Sigma_{MSOA} + w_{12}\mathbb{I}_n & \beta_2^2 \Sigma_{MSOA} + w_2^2 \mathbb{I}_n \end{pmatrix}. \end{aligned}$$

□

Lemma A.5. *The answer to any predictive problem is a preditive distribution. The predictive distribution in its general form is usually the conditional distribution of the predictive target given the observed data.* $LEB^* = (LEB_1(x_1), \ldots, LEB_1(x_q), LEB_2(x_1), \ldots, LEB_2(x_q))^\top$ *be the predictive target and* LEB *be the vector of observed data, then the predictive distribution is formally expressed as* $[LEB^*|LEB]$

Theorem A.6. *Let* $LEB^* = (LEB_1(x_1), \ldots, LEB_1(x_q), LEB_2(x_1), \ldots, LEB_2(x_q))^\top$*; the predictive distribution for* LEB^**, i.e. its conditional distribution given the data, is multivariate Gaussian with mean*

$$\alpha \oplus \mathbb{1}_{q\times 1} + D^\top \Sigma_{LEB}^{-1}(LEB - \alpha \oplus \mathbb{1}_{n\times 1}), \quad (7)$$

and covariance matrix

$$\Sigma_{LEB^*} - D^\top \Sigma_{LEB}^{-1} D. \quad (8)$$

In (8), the (h, h')*-th element of* Σ_{LEB^*} *is given by* $(\Sigma_{LEB^*})_{hh'} = \tau^2 \exp\{-\|x_h - x_{h'}\|/\delta\}$*. Also,*

$$D = \begin{pmatrix} D_1 \\ D_2 \end{pmatrix}$$

where D_i *is the* $n \times q$ *matrix whose* h*-th column is* $(d_1(x_h), \ldots, d_n(x_h))$*, and* $d_j(x_h) = \beta_i^2 \tau^2 \int_{MSOA_j} \exp\{-\|x_h - x\|/\delta\}\, dx$*.*

Proof. According the Lemma A.5, the predictive distribution is given as $[LEB^*|LEB]$. And using the properties of conditional distribution in Lemma A.3, the $[LEB^*|LEB]$ is follows a multivariate Gaussian with mean

$$\alpha \oplus \mathbb{1}_{q\times 1} + D^\top \Sigma_{LEB}^{-1}(LEB - \alpha \oplus \mathbb{1}_{n\times 1}), \tag{9}$$

and covariance matrix

$$\Sigma_{LEB^*} - D^\top \Sigma_{LEB}^{-1} D. \tag{10}$$

In (8), the (h, h')-th element of Σ_{LEB^*} is given by $(\Sigma_{LEB^*})_{hh'} = \tau^2 \exp\{-\|x_h - x_{h'}\|/\delta\}$. Also,

$$D = \begin{pmatrix} D_1 \\ D_2 \end{pmatrix}$$

where D_i is the $n \times q$ matrix whose h-th column is $(d_1(x_h), \ldots, d_n(x_h))$, and $d_j(x_h) = \beta_i^2 \tau^2 \int_{MSOA_j} \exp\{-\|x_h - x\|/\delta\}\, dx$. □

B Map of the Observed IMD and LEB

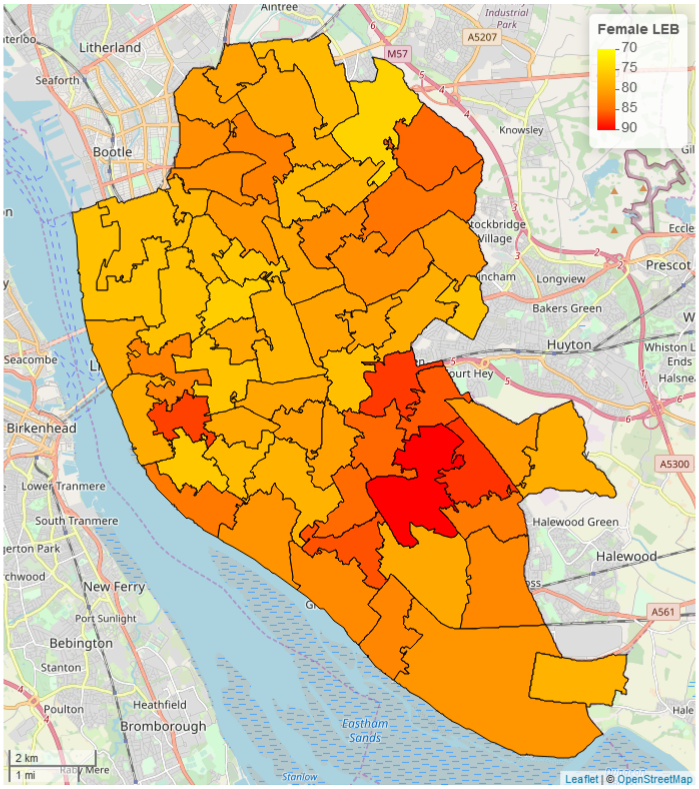

Figure B.1: Map of the observed female life expectancy at birth (LEB)

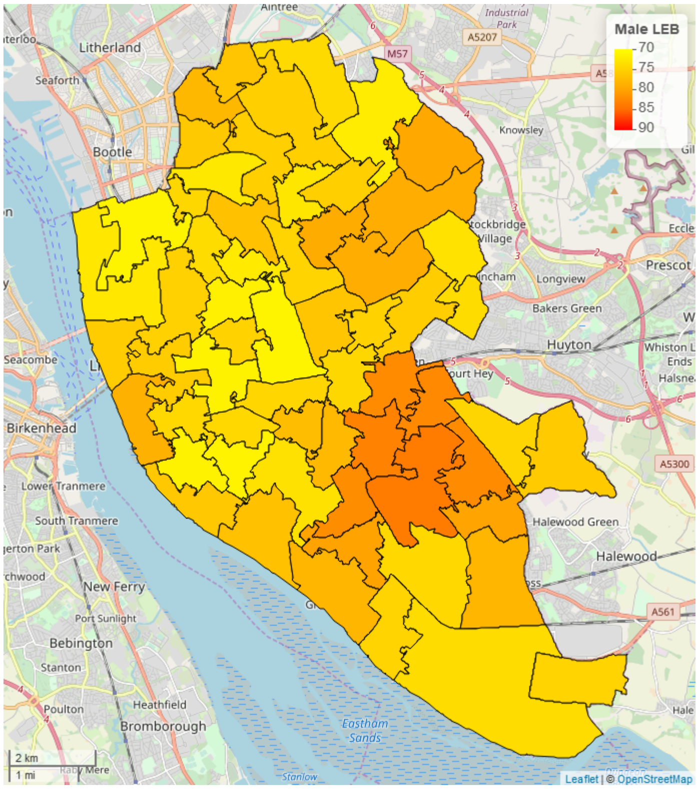

Figure B.2: Map of the observed male life expectancy at birth (LEB)

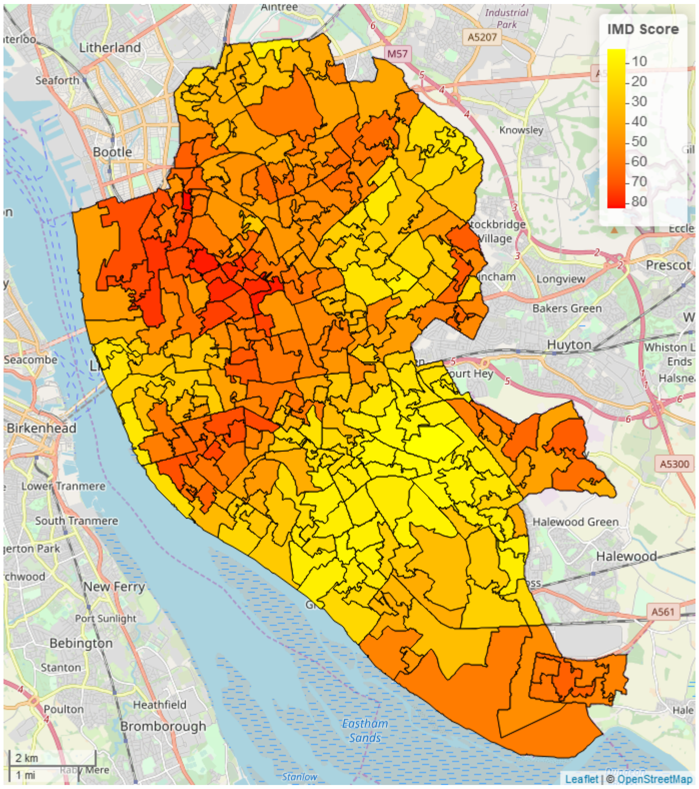

Figure B.3: Map of the observed index of multiple deprivation (IMD).

C Map of the Liverpool, MSOA, LSOA and Ward

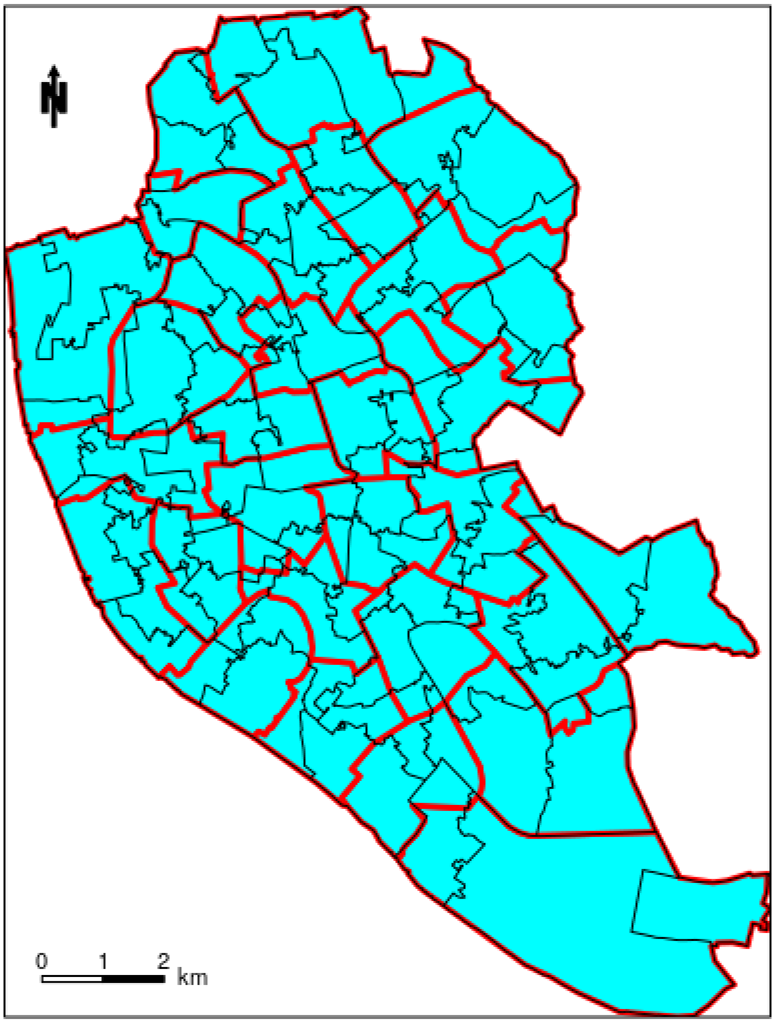

Figure C.1: Maps of Liverpool wards with MSOA boundaries overlayed. Red lines are the ward boundaries while black lines are the MSOA boundaries

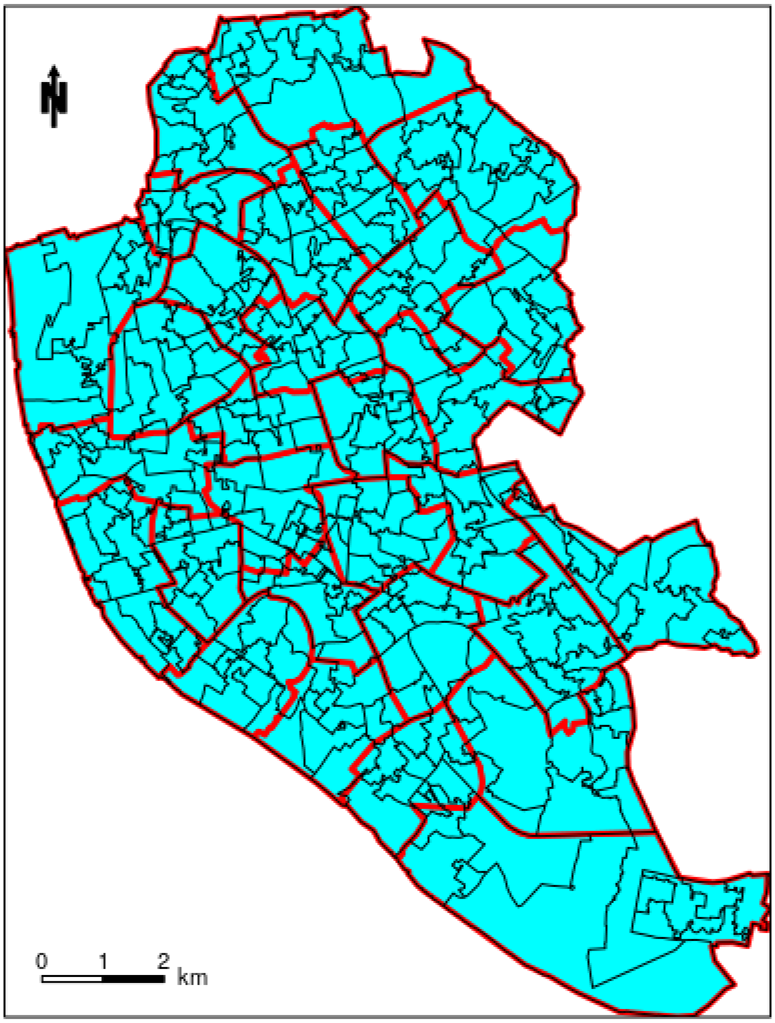

Figure C.2: Maps of Liverpool wards with LSOA boundaries overlayed. Red lines are the ward boundaries while black lines are the LSOA boundaries
